# Intracranial Pressure Reduction Is Associated with Mitochondrial OPA1 and Cytochrome c Release in the Retinas of AQP1-Null Mice

**DOI:** 10.3390/brainsci16050470

**Published:** 2026-04-28

**Authors:** Zheng Zhang, Shen Wu, Kegao Liu, Jingxue Zhang, Qian Liu, Ningli Wang, Hai Xue

**Affiliations:** 1 Beijing Tongren Eye Center, Beijing Ophthalmology and Visual Sciences Key Laboratory, Beijing Tongren Hospital, Capital Medical University, Beijing 100730, China; zhangzheng9819@163.com (Z.Z.);; 2 Beijing Institute of Ophthalmology, Beijing Tongren Hospital, Capital Medical University, Beijing 100005, China; 3 Department of Neurosurgery, Beijing Tiantan Hospital, Capital Medical University, Beijing 100070, China; 4 China National Clinical Research Center for Neurological Diseases, Beijing 100070, China

**Keywords:** intracranial pressure, mitochondria, optic atrophy-1 (OPA1), cytochrome c, aquaporin 1

## Abstract

Background: Recent studies strongly suggest that low intracranial pressure (ICP) may be involved in the pathogenesis of glaucomatous optic neuropathy. As retinal ganglion cells (RGCs) are highly susceptible to mitochondrial dysfunction, mitochondrial injury may be associated with optic neuropathy related to reduced ICP. In this study, aquaporin-1 (AQP1)-null mice were used to investigate whether reduced ICP is associated with alterations in mitochondrial structure and the release of optic atrophy type 1 (OPA1) and cytochrome c from mitochondria. Methods: Intraocular pressure (IOP) and ICP were measured in AQP1-null mice, and mitochondrial structural changes were examined using transmission electron microscopy (TEM). Total OPA1 and cytochrome c protein levels were evaluated using immunocytochemistry and Western blotting. Cytosolic and mitochondrial fractions were extracted from retinal tissues, and the subcellular distribution of OPA1 and cytochrome c was further analyzed by Western blotting. Bax and Bcl-2 expression levels were also detected. Results: TEM revealed mitochondrial fission, matrix swelling, and abnormal cristae depletion in the retinas of 1-, 3-, and 6-month-old AQP1-null mice. Morphometric quantification further confirmed significantly reduced mitochondrial length across all age groups and increased mitochondrial width at 1 and 6 months in AQP1-null mice compared with wild-type controls. Decreased retinal OPA1 immunoreactivity and protein expression were observed across all age groups of AQP1-null mice compared with age-matched C57BL/6 control mice. Subcellular fractionation showed increased mitochondrial release of OPA1 (at 3 and 6 months) and cytochrome c (at 1, 3, and 6 months) in the retinas of AQP1-null mice. Altered Bax expression was also detected in the retinas of AQP1-null mice with reduced ICP at all examined ages. Conclusions: Mitochondrial ultrastructural abnormalities, including fission and cristae depletion, altered OPA1 distribution, increased mitochondrial release of OPA1 and cytochrome c, and upregulated Bax expression were observed in the retinas of AQP1-null mice with reduced ICP. These concurrent changes indicate a close association between reduced ICP and retinal mitochondrial dysfunction. Maintaining mitochondrial integrity may therefore serve as a potential protective strategy against optic nerve degeneration in patients with chronic low ICP.

## 1. Introduction

Glaucoma is one of the major causes of irreversible blindness worldwide [1]. Primary open-angle glaucoma (POAG), the most prevalent subtype, is marked by the progressive degeneration of retinal ganglion cells (RGCs) and their axons within the optic nerve, accompanied by optic nerve head cupping and a corresponding visual field loss [2]. While elevated intraocular pressure (IOP) is recognized as a key risk factor for POAG development, studies of patients with normal-pressure glaucoma (NTG) have indicated that other contributing factors may also be involved [3,4]. Notably, the difference in translaminar cribrosa pressure—defined as the difference between the IOP and the pressure in the subarachnoid space (SAS) along the optic nerve—plays a critical role at the optic nerve head [5]. Recent research has suggested an association between low pressure behind the cribrosa and the pathogenesis of glaucomatous optic neuropathy [6]. Researchers have proposed that reduced intracranial pressure (ICP) might be linked to cerebrospinal fluid (CSF) circulatory dysfunction, which could potentially impair neurotoxin clearance along the optic nerves; the accumulation of bioactive substances may therefore exert adverse effects on RGCs and their axons [7]. However, the exact pathophysiological link between reduced ICP and RGC death remains unclear.

Neurons, including RGCs, have high energy requirements to maintain their normal function, and mitochondria are essential organelles for cellular energy metabolism. Pathological mitochondrial changes have been frequently observed in the retina and optic nerve of animal models with elevated IOP, yet such changes in low-ICP models have been rarely reported. Mitochondrial fission and fusion are dynamic processes that facilitate the mixing of metabolites and mitochondrial DNA (mtDNA), and regulate organelle shape, quantity, and bioenergetic function [8]. This balance between fission and fusion is governed by a family of dynamin-related GTPases, with Fzo1 and Mgm1 serving as key mediators. The mammalian homolog of Mgm1 is optic atrophy type 1 (OPA1), which is involved in multiple processes related to mitochondrial inner membrane fusion; mutations in OPA1 are associated with various human neurodegenerative diseases [9].

OPA1 is widely expressed in the soma and axons of RGCs and horizontal cells [10,11], and emerging evidence suggests an association between OPA1 gene polymorphisms and POAG [12]. Elevated IOP has been shown to alter OPA1 expression, which is associated with mitochondrial fission and cytochrome c release [13,14]. Additionally, OPA1 release during mitochondrial fission is linked to apoptotic cell death [15]. However, whether a long-term ICP reduction is associated with changes in retinal OPA1 expression and distribution, and whether such changes are related to apoptotic processes, remains unclear. We explored these questions in an in vivo model of chronic intracranial hypotension by evaluating the changes in mitochondrial morphology and OPA1 expression in the retinas of AQP1-deficient mice—a strain that spontaneously develops reduced ICP [16]. We hypothesized that a long-term reduction in the ICP may be associated with mitochondrial morphological changes in RGCs linked to an altered fission-fusion balance.

## 2. Materials and Methods

### 2.1. Animals

All procedures involving animals were performed in accordance with the ARVO Statement for the Use of Animals in Ophthalmic and Vision Research. Adult 1-, 3-, and 6-month-old male AQP1-null mice (Nanjing Biomedical Research Institute of Nanjing University) and age-matched male C57BL/6 mice were housed in temperature-controlled rooms with a 12 h light/dark cycle and were provided standard food and water ad libitum. All experimental mice were randomly allocated into experimental groups by age before tissue collection.

### 2.2. Generation of AQP1-Null Mice

AQP1-knockout mice were generated using the CRISPR/Cas9 system. First, two sgRNAs targeting exon 1 of AQP1 were constructed and transcribed in vitro. The Cas9 mRNA and sgRNA were subsequently coinjected into zygotes, which were then transferred into the oviducts of pseudopregnant ICR females at 0.5 days postcoitus (dpc). F0 mice were delivered 19–21 days after embryo transfer. All the offspring of ICR females (F0 mice) were identified by PCR and tail DNA sequencing, and positive F0 mice were genotyped using previously established protocols. Finally, F0 mice were crossed with C57BL/6J mice to generate F1 heterozygous mice. Offspring from crosses of heterozygous mice were genotyped by PCR, and homozygous knockout mice were obtained for subsequent experiments.

### 2.3. ICP Measurement

The ICP was measured using a BIOPAC Systems MP150 workstation (BIOPAC Systems Co., Goleta, CA, USA). The mice were anesthetized with a mixture of ketamine (80 mg/kg) and xylazine (10 mg/kg) and immobilized in the prone position in a stereotactic guide instrument. A dorsal midline incision was made over the skull and the upper cervical spine, and the cranial sutures were exposed. A burr hole of 1 mm in diameter was made with a dental drill in the left parietal bone above the left lateral ventricle, using coordinates 1 mm lateral and 1 mm caudal to the bregma. The dura mater was gently punctured with a 30-gauge needle to ensure egress of the CSF and entrance into the subarachnoid space (SAS). A 1.6F pressure catheter (4.8 mm tube length below the skull; Scisense, Inc., London, ON, Canada) was inserted vertically deep into the SAS to measure the ICP. The ICP measured in mmHg was continuously monitored.

### 2.4. IOP Measurement

The IOP was measured with a rebound tonometer optimized for mouse use (Tonolab Colonial Medical Supply, Franconia, NH, USA). Anesthetized mice were placed on an adjustable stand, and the tail was restrained with adhesive tape. The probe tip was aligned with the optical axis of the eye at a distance of 1-2 mm without local corneal anesthesia. Five consecutive IOP readings were recorded and averaged for each eye [17].

### 2.5. Tissue Preparations

Deeply anesthetized mice were transcardially perfused with 0.01 M PBS (pH 7.4, 37 °C), followed by 4% paraformaldehyde. The eyes were enucleated and completely immersed in 4% paraformaldehyde for 1 h, followed by cryoprotection in 30% sucrose in PBS. The eyes were cryopreserved in OCT (Sakura Finetek USA, Inc., Torrance, CA, USA) and stored at −80 °C until use.

### 2.6. Immunohistochemical Analyses

The samples were longitudinally sectioned into 7-μm-thick slices using a cryotome (Leica Microsystem, Wetzlar, Germany) set at −22 °C. The slices were rinsed with PBS 3 times for 5 min at room temperature (RT). The tissue sections were incubated with 5% bovine serum albumin for 30 min at RT to prevent nonspecific background staining and then incubated with a primary polyclonal rabbit antibody against cytochrome c (1:500; Abcam, Cambridge, MA, USA) and a mouse monoclonal antibody against OPA1 (1:200; Abcam, Cambridge, MA, USA) overnight at 4 °C. After the sections were rinsed to remove the excess primary antibody, the sections were incubated in the dark with Alexa Fluor 488-conjugated anti-mouse (1:1000) or Alexa Fluor 594-conjugated anti-rabbit (1:1000) secondary antibodies for 1 h at RT and then washed with PBS. The sections were counterstained with nucleic acid stain (Hoechst 33342 1 μg/mL; Invitrogen–Molecular Probes, Eugene, OR, USA) in PBS. Images of tissue sections were captured using a fluorescence microscope (DM 4000B; Leica, Wetzlar, Germany).

### 2.7. Transmission Electron Microscopy

The mice were deeply anesthetized, transcardially perfused with 0.01 M PBS (pH 7.4, 37 °C), and then fixed with a 2% paraformaldehyde and 2.5% glutaraldehyde mixture in PBS. Posterior eye segments were postfixed with 1% buffered osmium tetroxide, dehydrated in graded ethanol solutions and embedded in Epon 812. The area centered on the retina-choroid-sclera complex was thin-sectioned and stained with 5% uranyl acetate and lead citrate. Ultrathin sections were mounted on mesh nickel grids and examined using a Hitachi H-7650 electron microscope (Hitachi High-Tech, Tokyo, Japan).

The mitochondrial length and width were quantified using ImageJ software (Version 1.54f; National Institutes of Health, Bethesda, MD, USA). Three male mice were included in each group. For quantification, a total of 50 intact mitochondria with clear double membranes were randomly selected and measured per group. Mitochondrial length was defined as the maximum distance along the major axis between the two farthest poles of each mitochondrion. Mitochondrial width was defined as the maximal transverse diameter measured perpendicularly to the major axis at the midpoint of the mitochondrial length. All measurements were performed in a blinded manner.

### 2.8. Western Blot Analysis

Retinas were isolated from AQP1-null and C57BL/6 mice at 1, 3, and 6 months (*n* = 4 per group). Retinas from 4 mice were pooled and homogenized in ice-cold RIPA buffer (Sigma Aldrich, St. Louis, MO, USA) containing protease and phosphatase inhibitors. The resulting lysates were centrifuged at 12,000× *g* for 15 min at 4 °C, and the protein concentration in the supernatant was measured thereafter. Proteins were separated via sodium dodecyl sulfate-polyacrylamide gel electrophoresis (SDS-PAGE) on 7% gels, with 10 μg of protein loaded into each lane. Following electrophoresis, the proteins were transferred onto a 0.2 μm pore size polyvinylidene difluoride (PVDF) membrane (Bio-Rad, Richmond, CA, USA).

The PVDF membrane was blocked with 5% nonfat dry milk dissolved in TBST buffer (0.05% Tween-20 in PBS) and then incubated overnight at 4 °C with the following primary antibodies: monoclonal mouse anti-OPA1 antibody (1:1000; Abcam, Cambridge, MA, USA), monoclonal rabbit anti-cytochrome c antibody (1:1000; Abcam, Cambridge, MA, USA), monoclonal mouse anti-Bax antibody (1:1000; Abcam, Cambridge, MA, USA), monoclonal rabbit anti-Bcl-2 antibody (1:2000; Abcam, Cambridge, MA, USA), monoclonal mouse anti-actin antibody (1:2000; CWBIO, Beijing, China), and monoclonal rabbit anti-GAPDH antibody (1:2000; CWBIO, Beijing, China). After the incubation with the primary antibodies, the membrane was rinsed thoroughly with 0.05% Tween-20/PBS and then incubated with peroxidase-conjugated secondary antibodies (goat anti-mouse or goat anti-rabbit, both at 1:2000; CWBIO, Beijing, China). Protein bands were visualized using enhanced chemiluminescence (ECL) detection reagents (CWBIO). Images were analyzed using Quantity One software (Version 4.6.8; Bio-Rad Laboratories, Hercules, CA, USA), and band densities were normalized to actin or GAPDH as an internal reference. Western blot analyses were performed in three independent biological replicates (*n* = 3), and band intensities were quantified based on these repeated experiments for statistical analysis.

Cytosolic and mitochondrial fractions were isolated from freshly harvested retinas (4 retinas per group) using differential centrifugation with a Mitochondrial Isolation Kit (Pierce Biotechnology, Rockford, IL, USA) to determine the subcellular localization of OPA1 and cytochrome c. Briefly, retinal tissues were immediately homogenized in reagent A using a glass–Teflon Potter–Elvehjem homogenizer (Kimble Chase, Vineland, NJ, USA), mixed with an equal volume of reagent C, and centrifuged at 700× *g* for 10 min at 4 °C. The supernatant was collected and further centrifuged at 12,000× *g* for 15 min at 4 °C; the resulting supernatant was designated as the cytosolic fraction. For the mitochondrial fraction, the mitochondrial pellet obtained from the second centrifugation was lysed with 2% CHAPS in Tris-buffered saline and centrifuged again at 12,000× *g* for 15 min at 4 °C, with the supernatant collected as the mitochondrial fraction.

Western blot analysis was performed on the isolated fractions using the same protocol described above. Equal protein loading was verified by reprobing the membranes containing the proteins in the cytosolic fractions with a monoclonal mouse anti-actin antibody (1:2000; CWBIO, Beijing, China) and the membranes containing the proteins in the mitochondrial fractions with a polyclonal rabbit anti-VDAC antibody (1:1000; Abcam, Cambridge, MA, USA). Effective separation of the two fractions was confirmed by the absence of significant cross-contamination: negligible VDAC signals were observed in cytosolic fractions, and minimal actin signals were detected in mitochondrial fractions [13].

### 2.9. Statistical Analysis

The experiments were repeated at least three times. All image acquisition and quantitative analyses were performed by investigators who were blinded to the genotypes and grouping information of the samples. Statistical analyses were performed using a commercially available software system (SPSS for Windows, version 21.0; IBM-SPSS, Chicago, IL, USA). The data are presented as the means ± SDs. A paired *t*-test was used for comparisons between baseline and follow-up values. An independent *t*-test was used to compare the age-matched study group and the control group. Comparisons of three age groups of AQP1-null mice or C57BL/6 mice were evaluated using one-way analysis of variance (ANOVA) followed by the Bonferroni post hoc test. *p* < 0.05 was considered statistically significant.

## 3. Results

### 3.1. Animal Physiology

**Figure 1:**
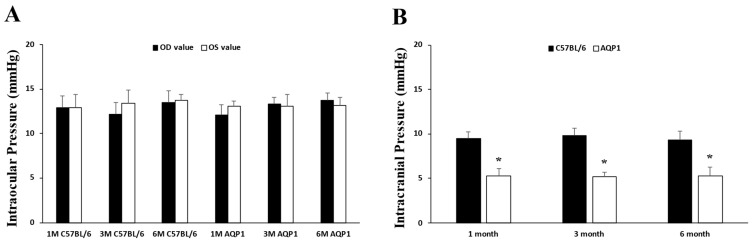
The average IOPs (**A**) and ICPs (**B**) in 1-, 3-, and 6-month-old C57BL/6 mice and age-matched AQP1-null mice. OD = oculus dexter (right eye); OS = oculus sinister (left eye). Compared with age-matched C57BL/6 mice, AQP1-null mice showed a significantly lower ICP. Error bar, SD. * Significant at *p* < 0.001 compared with age-matched C57BL/6 mice; *n* = 6 mice per group.

The IOP was measured monthly. The final IOP of adult 1-, 3-, and 6-month-old male C57BL/6 mice was shown in Figure 1. Averages values of the final IOPs did not differ significantly between the age-matched AQP1-null mice and C57BL/6 mice (12.6 ± 0.8 vs. 12.9 ± 0.8 mmHg in the 1-month matched groups; 13.2 ± 0.7 vs. 12.8 ± 0.7 mmHg in the 3-month matched groups; 13.5 ± 0.8 vs. 13.6 ± 0.9 mmHg in the 6-month matched groups; all *p* > 0.332). Compared with age-matched C57BL/6 mice, AQP1-null mice had significantly lower ICP (5.3 ± 0.9 vs. 9.5 ± 0.7 mmHg in the 1-month matched groups; 5.2 ± 0.6 vs. 9.8 ± 0.9 mmHg in the 3-month matched groups; 5.3 ± 1.0 vs. 9.4 ± 0.9 mmHg in the 6-month matched groups; all *p* < 0.001).

### 3.2. Mitochondrial Structural Alterations in the Retinas of Mice with Reduced ICP

**Figure 2:**
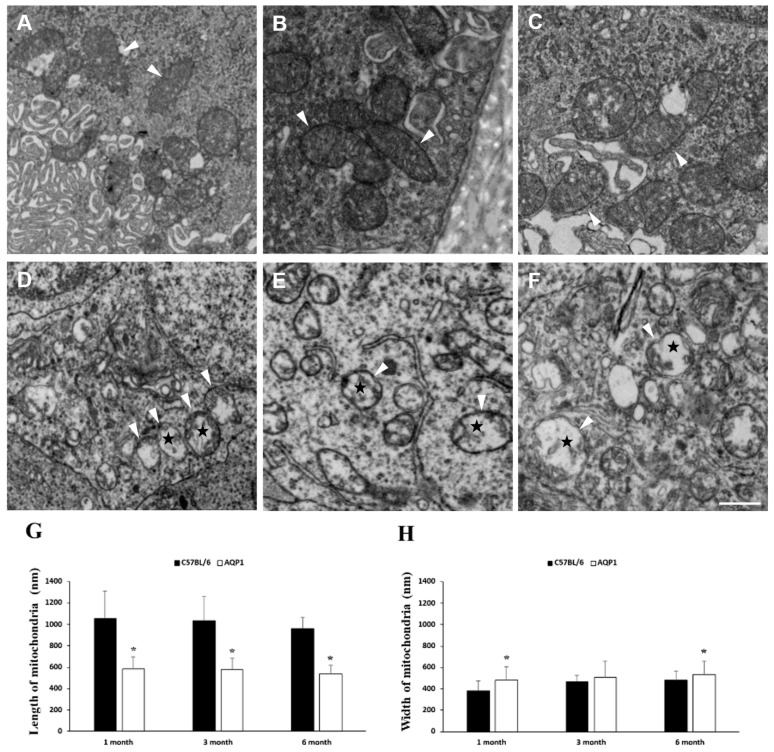
Ultrastructural changes in mitochondria in the retina. C57BL/6 mice aged 1 (**A**), 3 (**B**), and 6 months (**C**) exhibited elongated mitochondria (arrowheads) of various lengths with normal matrix and membrane structures. In contrast, mitochondria (arrowheads) from age-matched AQP1-null mice with a reduced ICP aged 1 (**D**), 3 (**E**), and 6 months (**F**) appeared smaller and rounded, with marked matrix swelling (asterisks). Scale bars: (**A**–**F**) 1 μm. (**G**) Quantitative analysis of mitochondrial length in the retina. (**H**) Quantitative analysis of mitochondrial width in the retina. Each group contained mitochondria from 3 independent mice (*n* = 50 mitochondria per group). Error bars, SDs. * Significant at *p* < 0.05 compared with age-matched C57BL/6 mice.

Mitochondrial structure was compared between the retinas of C57BL/6 mice with normal ICP and AQP1-null mice with reduced ICP. As shown in Figure 2, transmission electron microscopy (TEM) revealed that the retinas of 1-, 3-, and 6-month-old C57BL/6 mice contained classic elongated tubular mitochondria of various lengths (Figure 2A–C). In contrast, the retinas of age-matched AQP1-null mice exhibited small, rounded mitochondria with swollen matrices and reduced cristae density (Figure 2D–F).

The mitochondrial length was significantly shorter in AQP1-null mice than in age-matched C57BL/6 controls (581.9 ± 111.7 vs. 1055.5 ± 254.0 nm at 1 month; 578.2 ± 102.0 vs. 1034.7 ± 225.1 nm at 3 months; 537.1 ± 81.3 vs. 959.8 ± 106.4 nm at 6 months; all *p* < 0.001; Figure 2G). Significant increases in the mitochondrial width were observed in 1- and 6-month-old AQP1-null mice compared with age-matched wild-type mice (482.7 ± 122.8 vs. 379.3 ± 91.2 nm at 1 month; 530.5 ± 127.0 vs. 479.7 ± 86.7 nm at 6 months; all *p* < 0.022; Figure 2H).

### 3.3. Total OPA1 Protein Level in the Retinas of Mice with Reduced ICP

**Figure 3:**
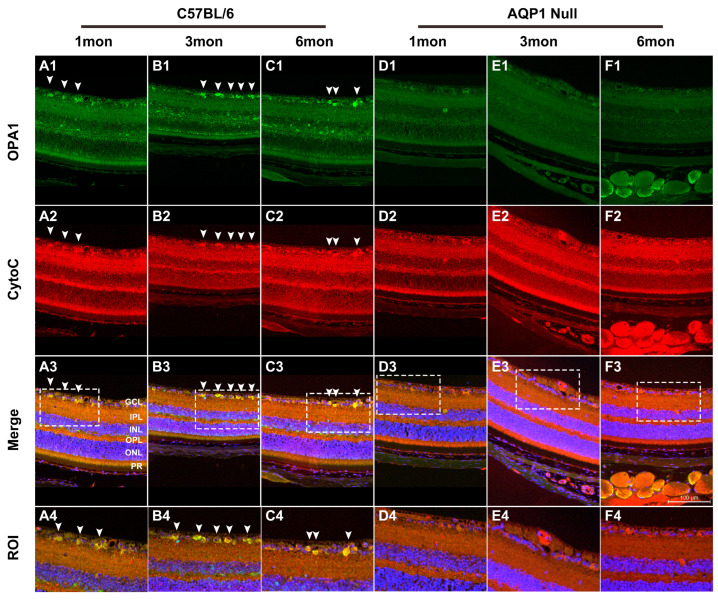
OPA1 and cytochrome c immunohistochemistry of retinas from 1-, 3-, and 6-month-old C57BL/6 mice and age-matched AQP1-null mice. OPA1 (**A1**–**F1**, green) and cytochrome c (**A2**–**F2**, red), merged images (**A3**–**F3**), and higher-magnification images of regions of interest (ROIs, **A4**–**F4**). The dashed squares in the merged images (**A3**–**F3**) indicate the specific regions of interest (ROIs) that are magnified in the corresponding (**A4**–**F4**) images, allowing for clearer observation of OPA1 and cytochrome c localization in target retinal cells. The purple, yellow, and orange colors in the images represent the colocalization or overlapping of different markers: purple indicates the overlap of cytochrome c (red) and Hoechst 33,342 (blue), showing the localization of cytochrome c around the nucleus; yellow indicates the colocalization of OPA1 (green) and cytochrome c (red), suggesting the co-expression of OPA1 and cytochrome c in the same cellular compartment; orange is formed by a predominance of red (cytochrome c) with a small amount of green (OPA1) light overlap, reflecting the region where cytochrome c is the main marker and OPA1 is weakly expressed. The sections were counterstained with the nucleic acid stain Hoechst 33,342 (blue). Retinal layers, including the ganglion cell layer (GCL), inner plexiform layer (IPL), inner nuclear layer (INL), outer plexiform layer (OPL), outer nuclear layer (ONL), and photoreceptor layer (PR), are labeled in the merged images (**A3**). Arrowheads indicate the ROI cells in the images. OPA1 immunoreactivity was abundantly detected in the retinas of 1-, 3-, and 6-month-old C57BL/6 mice. In contrast, much lower OPA1 immunoreactivity was observed in the retinas of age-matched AQP1-null mice. Scale bar, 100 μm.

**Figure 4:**
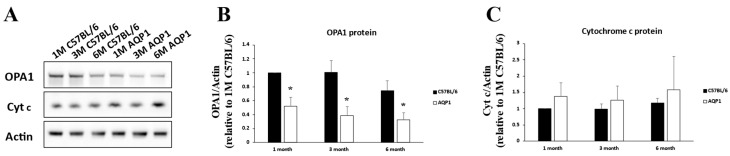
Changes in total OPA1 and cytochrome c expression in the retinas of C57BL/6 mice and age-matched AQP1-null mice. (**A**) The cytochrome c protein bands show the positions of the 12 kDa form of cytochrome c. The OPA1 protein bands show the positions of the 82 kDa form of OPA1. The relative intensity of chemiluminescence for each protein band was normalized using actin (~42 kDa) as the calibrator. (**B**) Compared with those in age-matched C57BL/6 mice, the levels of the OPA1 protein bands in 1-, 3-, and 6-month-old AQP1-null mice were significantly decreased. (**C**) The cytochrome c protein bands showed slight changes, but the difference was not significant. Each pooled sample contained retinas from 4 independent mice per group (*n* = 4 retinas per pool). Western blot analyses were performed in three independent experiments for statistical quantification. Error bars, SD. * Significant at *p* < 0.05 compared with age-matched C57BL/6 mice.

We examined retinal OPA1 expression in mice with reduced ICP by performing Western blotting and immunohistochemistry. OPA1 immunoreactivity was present in the ganglion cell layer (GCL), inner plexiform layer (IPL), inner nuclear layer (INL), outer plexiform layer (OPL), and photoreceptor layer (PR) in the retinas of 1-, 3-, and 6-month-old C57BL/6 mice (Figure 3A–C). Notably, AQP1 gene knockout decreased OPA1 immunoreactivity in all layers of the retinas of age-matched AQP1-null mice (Figure 3D–F). Cytochrome c immunoreactivity was present in the retinas of all groups. As shown in Figure 4, the OPA1 protein levels in the retinas of the 1-, 3-, and 6-month-old AQP1-null mice were significantly lower (0.52 ± 0.13, 0.39 ± 0.11, and 0.43 ± 0.05-fold, respectively) than those in the retinas of age-matched C57BL/6 mice (all *p* < 0.022). The cytochrome c protein levels did not differ significantly (all *p* > 0.19) between the retinas of 1-, 3-, and 6-month-old AQP1-null mice and those of age-matched C57BL/6 mice.

### 3.4. Mitochondrial OPA1 Release in the Retinas of Mice with Reduced ICP

**Figure 5:**
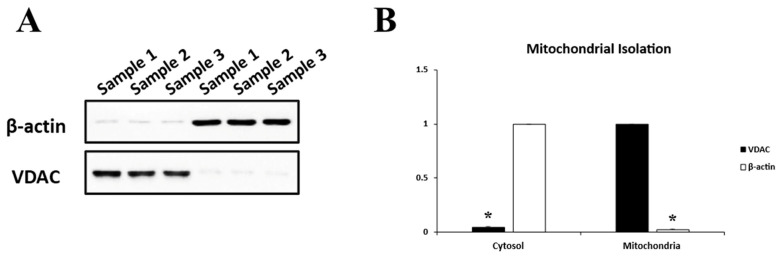
Verification of mitochondrial isolation. (**A**) The β-actin protein bands showed negligible signals when membranes containing the mitochondrial fraction were reprobed. The VDAC protein bands showed negligible signals when membranes containing the cytosolic fractions were reprobed. (**B**) Relative intensity of chemiluminescence for each protein band was normalized using actin (~42 KDa) as the cytosolic fraction calibrator and VDAC (~34 KDa) as the mitochondrial fraction calibrator. Each pooled sample contained retinas from 4 independent mice per group (*n* = 4 retinas per pool). There were 3 samples for the cytosolic fraction and 3 samples for the mitochondrial fraction. Error bars, SD. * Significant at *p* < 0.05 using Student’s *t*-test.

We explored the association between reduced ICP and alterations in the mitochondrial OPA1 distribution in the retinas of 1-, 3-, and 6-month-old AQP1-null mice by measuring relative OPA1 protein levels in cytosolic and mitochondrial fractions using Western blotting. The results were normalized to actin (cytosolic marker) and VDAC (mitochondrial marker). Effective separation of the two fractions was confirmed by minimal cross-contamination, as shown in Figure 5.

**Figure 6:**
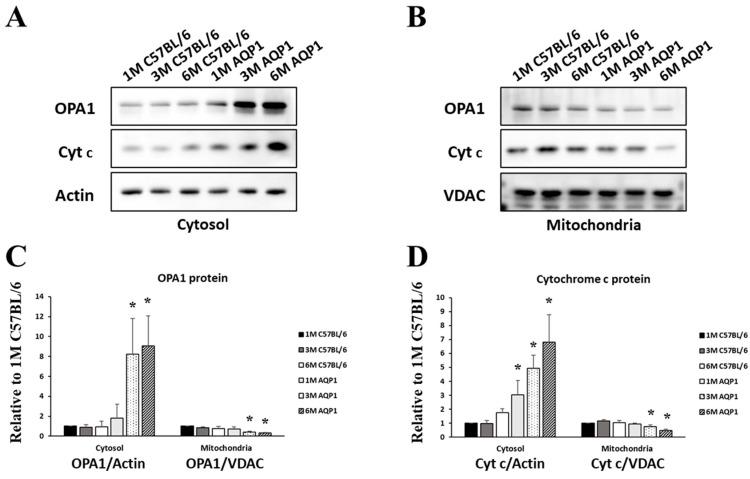
Associations between reduced ICP and mitochondrial redistribution of OPA1 and cytochrome c in the retina. (**A**,**B**) Western blot analysis of OPA1 levels in cytosolic and mitochondrial fractions from whole retinas. The cytochrome c protein bands indicate the positions of the 12 kDa form of cytochrome c in the cytosolic and mitochondrial fractions. The OPA1 protein bands indicate the positions of the 82 kDa form of OPA1. The relative intensity of chemiluminescence for each protein band was normalized using actin (~42 kDa) as the cytosolic fraction calibrator and VDAC (~34 kDa) as the mitochondrial fraction calibrator. (**C**) Cytosolic OPA1 band intensity was significantly increased in 3- and 6-month-old AQP1-null mice compared with age-matched C57BL/6 controls. In contrast, mitochondrial OPA1 band intensity was significantly decreased in 3- and 6-month-old AQP1-null mice. (**D**) Cytosolic cytochrome c band intensity was significantly higher in all AQP1-null mice than in age-matched C57BL/6 controls. However, mitochondrial cytochrome c band intensity was significantly decreased in 3- and 6-month-old AQP1-null mice. Each pooled sample contained retinas from 4 independent mice per group (*n* = 4 retinas per pool). Western blot analyses were performed in three independent experiments for statistical quantification. Error bars, SDs. * Significant at *p* < 0.05 compared with age-matched C57BL/6 mice.

As shown in Figure 6, compared with those of age-matched C57BL/6 mice, cytosolic OPA1 expression levels were significantly increased in the retinas of 3- and 6-month-old AQP1-null mice (8.78 ± 1.66 and 11.87 ± 5.82-fold, respectively; all *p* < 0.024; Figure 6A,C). In contrast, mitochondrial OPA1 expression levels were markedly decreased in the retinas of 3- and 6-month-old AQP1-null mice compared with age-matched controls (0.48 ± 0.13 and 0.45 ± 0.08-fold, respectively; all *p* < 0.026; Figure 6B,C).

Compared with age-matched C57BL/6 mice, cytosolic cytochrome c expression levels were significantly increased in the retinas of 1-, 3-, and 6-month-old AQP1-null mice (3.02 ± 1.04, 5.38 ± 1.92, and 3.87 ± 0.65-fold, respectively; all *p* < 0.044; Figure 6A,D). In addition, cytosolic cytochrome c expression was significantly higher in 6-month-old AQP1-null mice than in 1-month-old AQP1-null mice (ANOVA with a post hoc analysis; *p* = 0.008; Figure 6A,D). Conversely, mitochondrial cytochrome c expression (0.64 ± 0.09 and 0.47 ± 0.16-fold) was significantly lower in 3- and 6-month-old AQP1-null mice compared with age-matched controls (all *p* < 0.011; Figure 6B,D). Moreover, mitochondrial cytochrome c expression was significantly lower in 6-month-old AQP1-null mice than in 1-month-old AQP1-null mice (ANOVA with a post hoc analysis; *p* = 0.004; Figure 6B,D).

### 3.5. Bcl-2 and Bax Protein Expression in the Retinas of Mice with Reduced ICP

**Figure 7:**
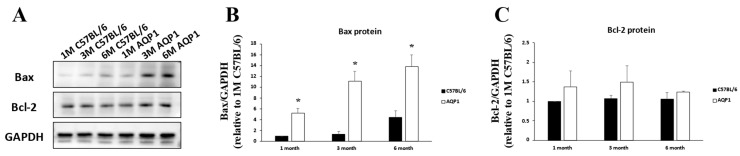
Association between reduced ICP and altered retinal Bax expression in AQP1-null mice. (**A**) The Bax protein bands show the positions of the 22 kDa form of Bax in the retinas of C57BL/6 mice and age-matched AQP1-null mice. The Bcl-2 protein bands indicate the positions of the 26 kDa form of Bcl-2. The relative chemiluminescence intensity for each protein band was normalized to that of GAPDH (36 kDa), which was used as the calibrator. (**B**) Bax expression was significantly higher in AQP1-null mice than in age-matched C57BL/6 controls. (**C**) The Bcl-2 protein levels showed no significant changes. Each pooled sample contained retinas from 4 independent mice per group (*n* = 4 retinas per pool). Western blot analyses were performed in three independent experiments for statistical quantification. Error bars, SD. * Significant at *p* < 0.05 compared with age-matched C57BL/6 mice.

In AQP1-null mice with reduced ICP, Bcl-2 expression remained unchanged across groups. Western blot analysis revealed that the Bax levels were significantly increased in the retinas of 1-, 3-, and 6-month-old AQP1-null mice (5.23 ± 0.86-, 9.69 ± 4.47-, and 3.22 ± 0.88-fold, respectively) compared with those in the retinas of age-matched C57BL/6 mice (all *p* < 0.003; Figure 7). In addition, Bax levels in the retinas of 3- and 6-month-old AQP1-null mice were significantly higher than those in 1-month-old AQP1-null mice (ANOVA with post hoc analysis; all *p* < 0.002; Figure 7).

## 4. Discussion

AQP1 is localized to the ventricular-facing membrane of the choroid plexus epithelium, playing a key role in cerebrospinal fluid (CSF) secretion [18]. In the present study, AQP1-null mice exhibited a 45% reduction in intracranial pressure (ICP) compared with age-matched C57BL/6 control mice, which is consistent with previous findings showing a 50% ICP reduction in AQP1-null mice [16]. Given that a chronic low ICP is closely associated with retinal neurodegeneration and optic neuropathy [3,4,6], AQP1-null mice represent a suitable animal model for studying optic neuropathy associated with a chronic low ICP and provide a reliable experimental basis for exploring the relationship between a low ICP and retinal mitochondrial dysfunction.

Our results showed substantial retinal changes in AQP1-null mice, including altered mitochondrial fission, abnormal crista depletion, decreased total OPA1 expression, mitochondrial OPA1 and cytochrome c release, and increased Bax protein expression. These changes were observed alongside a reduced ICP, with no significant IOP alterations, in AQP1-null mice.

RGCs are highly susceptible to mitochondrial dysfunction due to their high energy demands [19]. Our previous studies demonstrated that a reduced ICP disrupts axonal transport and alters the dynein and kinesin motor protein distribution [20,21], supporting the hypothesis that a bioenergetic impairment occurs under intracranial hypotension conditions and is closely associated with the mitochondrial changes observed in this study.

Mitochondrial morphology is governed by a balance between fission and fusion. Excessive fission can lead to mitochondrial DNA loss, respiratory defects, and increased reactive oxygen species levels [22]. Consistent with this paradigm, our ultrastructural observations using transmission electron microscopy indicated that retinal mitochondria in AQP1-null mice exhibited overt morphological abnormalities compared with age-matched C57BL/6 control mice. Morphometric quantification further confirmed that the mitochondrial length was significantly reduced in 1-, 3-, and 6-month-old AQP1-null mice, whereas the mitochondrial width was markedly increased in these mice at 1 and 6 months of age. Morphologically, these mitochondria also displayed fragmentation, matrix swelling, and a reduced cristae density. Collectively, the structural alterations and quantitative changes in mitochondrial dimensions indicate a shift toward excessive fission, which was observed concurrently with reduced ICP in the retinas of AQP1-null mice.

OPA1 is critical for maintaining mitochondrial morphology and optic nerve function, and is predominantly expressed in RGCs and optic nerve axons [10,11]. In AQP1-null mice, reduced OPA1 expression and its abnormal subcellular distribution (released from the mitochondria into the cytosol) were observed, alongside increased cytosolic cytochrome c levels. These changes were more pronounced in older AQP1-null mice, which is consistent with the well-documented age-related decline in mitochondrial function [23].

Bax, a proapoptotic Bcl-2 family member, mediates mitochondrial permeability and apoptotic signaling [24] and can contribute to the mitochondrial release of OPA1 and cytochrome c in the retina [25]. In AQP1-null mice, Bax protein expression was significantly increased compared with that in age-matched control mice, with no significant changes in Bcl-2 expression. This pattern is consistent with previous observations in glaucomatous models, where Bax upregulation occurs without alterations in Bcl-2 levels [26].

Age-related analyses showed that Bax expression and cytosolic OPA1/cytochrome c levels increased with age in AQP1-null mice, while total OPA1, total cytochrome c, and Bcl-2 expression remained stable. Mitochondrial cytochrome c levels were lowest in older-aged AQP1-null mice, suggesting that normal aging may synergize with a low ICP to shift the mitochondrial fission/fusion balance toward fission [23,27].

This study has several limitations. First, while AQP1-null mice are a well-established chronic low ICP model, we cannot completely rule out potential systemic effects of AQP1 deficiency independent of an ICP reduction. Generating stable, chronic low ICP models is technically challenging, and no targeted rescue or intervention experiments were performed in the present study. Future studies using independent low ICP models or ICP normalization in AQP1-deficient mice will help clarify the specific association between reduced ICP and retinal mitochondrial dysfunction. Second, we did not perform quantitative assessments of RGC loss (with specific markers), optic nerve axon morphometry, or functional visual tests. Thus, we cannot exclude the possibility that the observed mitochondrial changes reflect early cellular stress rather than progressive neuronal loss; additional in vivo functional assessments and quantitative neuropathology are needed to establish a direct disease-relevant chain of events. Third, subcellular fractionation quality was validated using the mitochondrial marker VDAC and the cytosolic marker β-actin, confirming minimal cross-contamination between fractions. The density gradient centrifugation protocol employed here is a well-established method in ocular mitochondrial research, consistent with the authoritative findings of Ju et al. [13]. While additional organelle markers or immunopurification were not applied in the current study to exclude minor membrane impurities, strict control of centrifugation parameters, buffer conditions and tissue processing procedures was performed to reduce non-specific contamination. Future mechanistic studies will adopt more rigorous purification strategies to improve mitochondrial purity and experimental reliability. Fourth, while transmission electron microscopy revealed mitochondrial ultrastructural changes, detailed characterization of mitochondrial dynamics, including fission–fusion regulatory proteins, would strengthen the mechanistic understanding. These aspects will be addressed in future studies to further clarify mitochondrial remodeling. Fifth, we observed alterations in OPA1 expression and mitochondrial–cytosolic redistribution in AQP1-null mice, but the mechanistic link between a low ICP and OPA1 regulation remains unclear, reflecting a correlation rather than direct causality. OPA1, a key regulator of mitochondrial fusion and cristae integrity, is modulated by post-translational modifications and upstream signals that may be affected by ICP, but the specific connecting molecular events are unknown. Future research will investigate OPA1-related regulatory factors (e.g., cleavage proteases, modifying kinases/phosphatases) and combine ICP rescue with interventions targeting the OPA1 pathway to clarify their directional relationship. Sixth, no direct evidence of RGC apoptosis (e.g., TUNEL staining or caspase activity) was obtained; TUNEL staining of 1-, 3-, and 6-month-old AQP1-null and wild-type mice yielded no positive signals. These observations indicate that the present findings reflect early apoptotic signaling rather than definitive, irreversible cell death. This may reflect early apoptotic events prior to DNA fragmentation, incomplete progression of Bax activation to irreversible apoptosis, or technical limitations. While the molecular findings are consistent with apoptotic activation, definitive confirmation requires future studies with additional time points and complementary apoptosis assays.

## 5. Conclusions

In summary, AQP1-null mice with reduced ICP exhibit mitochondrial fission, matrix swelling and cristae depletion; reduced OPA1 expression; abnormal subcellular distribution of OPA1 and cytochrome c; and elevated Bax levels. Maintaining mitochondrial integrity may therefore serve as a potential protective strategy against optic nerve degeneration in patients with chronic low ICP.

## Data Availability

The datasets generated and analyzed during the current study are not publicly available because they are associated with our additional ongoing unpublished work. However, all data supporting the findings of this study are available from the corresponding author upon reasonable request.
